# Hygiene Hints for Railway Travel

**Published:** 1893

**Authors:** E. A. Matthews

**Affiliations:** Carlinville, Ill.


### HALL'S JOURNAL OF HEALTH

HEALTH—THE POOR MAN'S RICHES, THE RICH MAN'S BLISS.

VOL. XL. SEPTEMBER–OCTOBER, 1893. NOS. 9 & 10.

### HYGIENE HINTS FOR RAILWAY TRAVEL.

We are fast becoming a nation of travellers. Year in and year out, we are restless enough, but when summer comes, then are our houses turned upside down and emptied of their contents, while the inmates roam about, now here—now there, like the dove of Noah, seeking in vain a resting place.

Of course there are reasons, many and good, for this strange restlessness on the part of our people. These I will not now discuss, but offer instead, a few suggestions upon the Hygiene of railway travel, and the best methods of overcoming its attendant evils.

A thoughtful observer says, "In view of the important part which railway travel plays in our lives as a people, and the risks run by our wives and children, it is curious that no one has seen fit to point out in language that even schoolboys could understand, the influence upon the physical being which so much persistent moving about must have."

All life upon the railway is a complete change from the ordinary, and even the strongest individual must be troubled by those inevitable horrors, shaking and dirt. In spite of all the refinements of the modern palace-car and sleeper, we cannot escape more or less of the jerk and jolt, the shaking and swaying of even our best roads.

The effect of these on brain and nerve is like the dreaded sea-sickness, and many a delicate person avoids the water to suffer from the same trouble on dry land.

When one is something of an invalid—not exactly sick, but "a little under the weather," the jar of the train is sure to act injuriously on the nerves. Many invalids we see starting on long journeys, alone, and with a recklessness truly astonishing.

If such persons must travel they should certainly take advice from a trusted and skilled physician and rely implicitly upon it. A few simple rules from such a source are greatly to be preferred to any self-doctoring, be the individual never so wise upon every day affairs. A dose of some tried sedative will often check the beginnings of a nervous attack,—a carefully prepared stimulant produces the needed "toning up" and no after troubles appear, such as are too apt to follow the medicines we choose for ourselves at such a time.

Even the most robust are exposed to many ills, unknown at home. Perhaps there is nothing more common than colds that come as the result of draughts. From open door, window or ventilator they are always ready to sweep down on the unwary.

In summer, when the system is all relaxed, perspiration at every pore, and the whole physique calling for coolness—up goes the windows, all rush to catch the delightful breeze—and the mischief is done. Then comes the severe cold, and all the attendant disagreeable consequences. The sleeping car is a regular nest for the growth of these ills, and no one can be secured against them. The only possible way to avoid them is by scrupulous care in dress and diet. All who travel must wear woolen garments next the skin. What is worn on the outside matters little if this one rule is obeyed. For adults this woolen underwear is essential ; for children to be without them is a crime.

Nowadays such articles are made of every grade and style, and no one who is able to pay the price of railway travel can be too poor to obtain them. The ideal dress for the delicate traveler or a child when in the sleeper, is a loose fitting wrapper of flannel, thick stockings (and knitted slippers when ready to step from dressing-room to berth), and over the ears and around the head an ample silk or woolen handkerchief. These need not be expensive—many a woman has saved herself from the earache or toothache, by the comfortable folds of an old dilapidated veil, or Shetland shawl.

Dirt is almost as great an evil as jolting in railway travel. It is impossible to keep clean. Examine the hands after even a short trip, and they will be found dirty ; and after a thorough washing there remains still a grime that has been absorbed by the skin so deeply as to be almost fixed. The open pores are clogged by these particles, just as corks stop the open bottle and thus we have the beginnings of kidney disease. Physicians are well acquainted with what is called "Railroad kidney," and its victims are railroad men, such as conductors, brakesmen, engineers and others.

Of course such results do not follow on ordinary journeys, but it is nevertheless true that those who already suffer from kidney disease are rendered much worse by travel on the rail.

Comfort may be secured at small cost and by an outlay of good judgment and forethought. A travelling bag is a necessity. In it should be, first—a sponge safely enclosed in a rubber bag. Keep the sponge damp, and when the face is hot or feverish, the cool moisture will awaken gratitude. Then add two or three soft old towels, some clean handkerchiefs, a tiny alcohol stove or lamp, with cups of tin in which to heat fluids, a flask of alcohol, a flask of strong, home-made beef stock, out of which to make beef tea or savory broth, some lemons, salt, sugar (safely kept in tin boxes), a bottle of some good fruit-salts that may be easily made into a tempting drink. In shawl straps carry rugs, pillows and a folding board.

This is a contrivance invented by an invalid and described in a recent medical journal for the benefit of those who have long felt the need of just such an article. "Take a piece of board eight inches wide, and three fourths of an inch in thickness. In length three feet and four inches, or long enough to rest an inch or so on each seat when it is placed across from one seat to another in a railway car. Saw it into two parts, and screw in two strap hinges, so that it will fold up. Carry it in the shawl strap and when opened with hinges on the under side, it forms a strong and perfect support for weary limbs. If several of these boards can be carried along, a very comfortable couch can be made for an invalid."

Of course such extra provision is not needed where all the party are well, but there are certain articles that prudence would always suggest, as accidents and sudden illness are so frequent. No doubt every family has a list of medicines that long usage has rendered essential.

The following is offered in cases of inexperience. Some of these are prescribed for emergencies and some for cases that seldom occur, but all have been advised by a skilled physician.

"A flask of good whiskey, one dozen cathartic pills, quinine pills (2 grains), compressed tablets of phenactine, used for headache and fever (5 grains), a menthol pencil, six ounces of soap liniment, a small quantity of adhesive plaster, a box of absorbent cotton, a roll of inch and half bandages, a dozen safety pins, a medicine measuring glass, a vial of camphor, a vial of spirits of ammonia, a vial of Sprudel salts, one bottle Jamaica ginger, one bottle acid phosphate, one bottle Sun cholera mixture or one bottle of Squibbs."

Of course no one would need all these remedies, but the long list is offered with the advice that in case of doubt, some good physician be asked to revise it. Sometimes a child is saved by a few drops of a remedy for bowel trouble, or for croup, and such an event would reward the traveler for numerous times of preparation and the bother of carrying the medicine.

Such articles must be placed in a bag, carefully marked, with name and dose. Let the medicine satchel be given into the care of a special person, and impress upon the bearer the great importance of the thing. Then be sure that it is never left behind or out of order, no matter what else is neglected.

One more hint—and that is,—never start on a journey with new shoes, new corsets, or any other new or stiff clothing. Save these vanities for quiet life, and take comfort in soft, old clothes, while " riding on the rail." MRS. E. A. MATTHEWS.

Carlinville, Ill.

